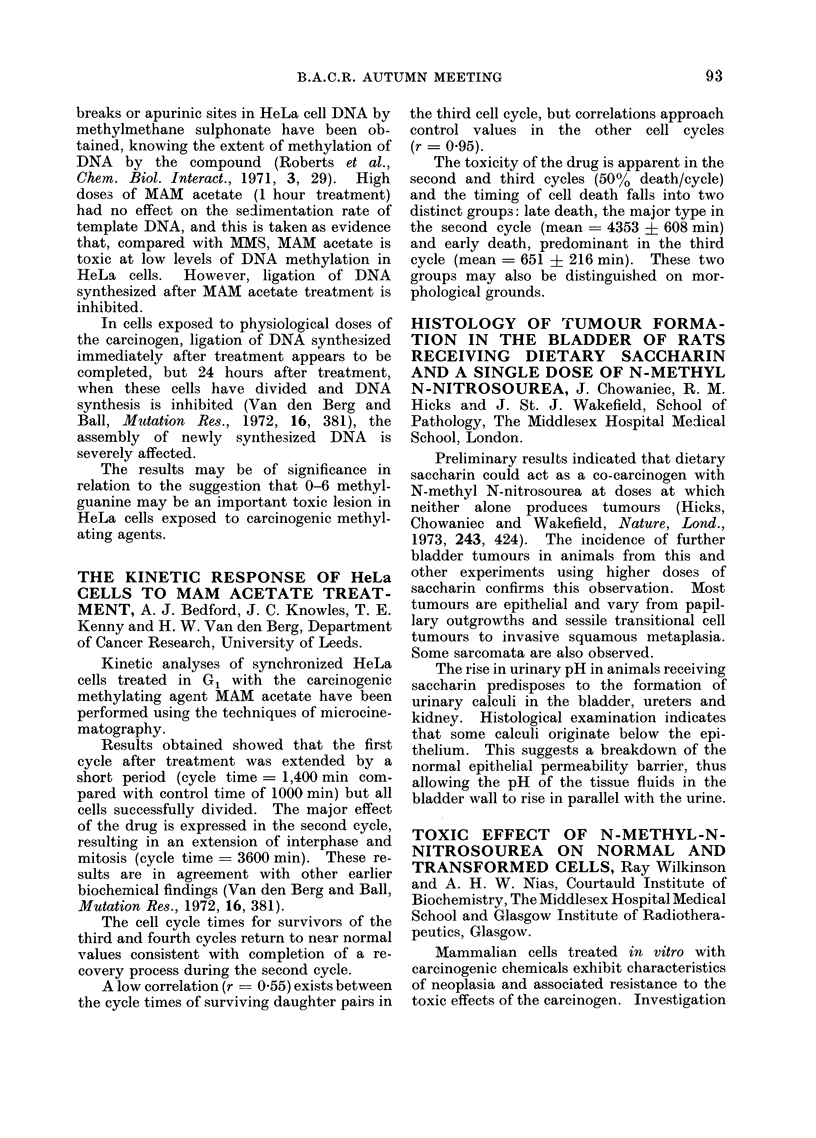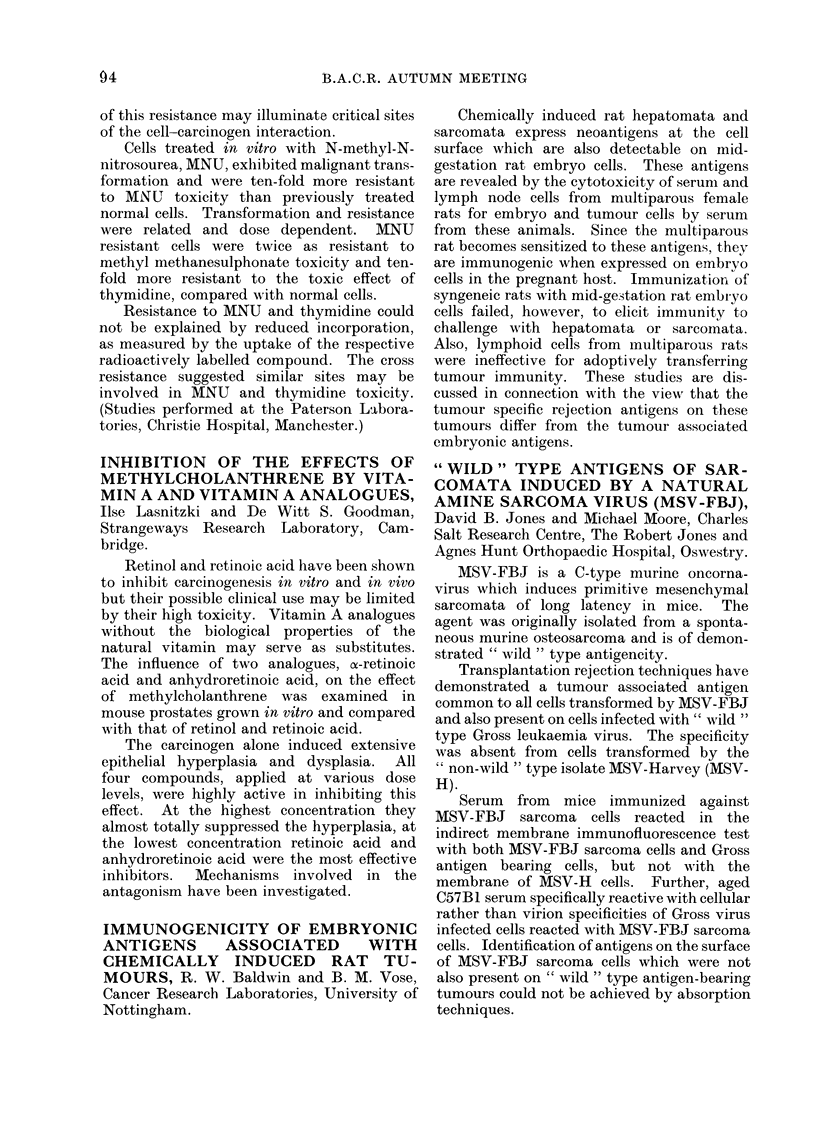# Proceedings: Toxic effect of N-methyl-N-nitrosourea on normal and transformed cells.

**DOI:** 10.1038/bjc.1974.19

**Published:** 1974-01

**Authors:** R. Wilkinson, A. H. Nias


**TOXIC EFFECT OF N-METHYL-N-NITROSOUREA ON NORMAL AND TRANSFORMED CELLS,** Ray Wilkinson and A. H. W. Nias, Courtauld Institute of Biochemistry, The Middlesex Hospital Medical School and Glasgow Institute of Radiotherapeutics, Glasgow.

Mammalian cells treated *in vitro* with carcinogenic chemicals exhibit characteristics of neoplasia and associated resistance to the toxic effects of the carcinogen. Investigation of this resistance may illuminate critical sites of the cell–carcinogen interaction.

Cells treated *in vitro* with N-methyl-N-nitrosourea, MNU, exhibited malignant transformation and were ten-fold more resistant to MNU toxicity than previously treated normal cells. Transformation and resistance were related and dose dependent. MNU resistant cells were twice as resistant to methyl methanesulphonate toxicity and ten-fold more resistant to the toxic effect of thymidine, compared with normal cells.

Resistance to MNU and thymidine could not be explained by reduced incorporation, as measured by the uptake of the respective radioactively labelled compound. The cross resistance suggested similar sites may be involved in MNU and thymidine toxicity. (Studies performed at the Paterson Laboratories, Christie Hospital, Manchester.)